# The Two Emergencies of Migrant-Related Policies in Italy During the First Wave of COVID-19: the Spread of the Virus and the Workforce Shortages

**DOI:** 10.1007/s12134-023-01042-8

**Published:** 2023-05-06

**Authors:** Nicola Montagna

**Affiliations:** School of Law, Middlesex University London, The Burroughs, London, NW4 4BT UK

**Keywords:** Bordering, COVID-19, Irregular migration, Migration policy, Quarantine ships, Floating hotspots

## Abstract

Italy was the first European country touched by COVID-19 and one of the most severely affected, with a death toll that overtook China’s by mid-March 2020. As a result, lockdown measures aiming to mitigate — and eventually interrupt — the spread of COVID-19 proliferated during the first wave of the pandemic. The vast majority of these concerned the resident population, regardless of their status or country of origin, and mainly involved the closure of public offices and proscription of private activities with the aim of reducing mobility and social and physical contacts. Only a few concerned the foreign population and arriving irregular migrants. This article analyses migrant-related policy measures taken by the Italian government during the first wave of the pandemic that aimed to prevent infection and reduce the impact of COVID-19 among the population. These measures addressed two emergencies: the spread of COVID-19 that hit the resident population hard, regardless of origin or nationality, and the workforce shortages in some key economic sectors with a high number of irregular migrant workers. The former aimed at containing the spread of the virus (sections 4 and 5) and targeted foreigners already residing in Italy as well as irregular migrants arriving along the Mediterranean route; the latter aimed at addressing workforce shortages (section 6) as a result of borders that were closed to external seasonal migration. This article is a contribution to the debate on changes to migration and migrant policy, and how these impacted on migration and foreign populations during the pandemic.

## Introduction

When COVID-19 broke out in the winter of 2019–2020, Italy was the first country in Europe to be severely affected. On 20th February “patient one” was identified in Codogno, a town of nearly 16,000 inhabitants in the province of Lodi in Lombardy. In the days that followed, other cases emerged in nearby villages, but locking these down did not prevent the infection from quickly spreading across the country, especially in the most industrialised and globally connected northern regions. By the beginning of March 2020, COVID-19 infections were out of control, and by mid-March the death toll had overtaken that of China, where the outbreak had begun in late 2019 (*The Guardian*, 19 March 2020). Italy, which was lauded by the *New York Times* (31 July 2020) for its ability to manage the pandemic better than countries such the UK and the USA, remained one of the places with the highest death toll over the summer, before a second wave hit the country.

Although neither newly arriving nor settled migrants had higher infection or mortality rates than the Italian population (Geraci et al. 2021), the association of the pandemic with migration — particularly with new arrivals — was shared by government and opposition parties and was used instrumentally according to their different agendas. For the right-wing opposition, the virus became a pretext for revitalising the anti-migration rhetoric that had favoured it during the previous electoral campaign; they targeted arriving migrants as plague-spreaders or as a privileged group given rights that Italians lacked. For the government, the pandemic was an opportunity to tighten national and EU borders. Although inflows did not reach the numbers of the previous years (2015–2018), the arrival of new migrants remained a priority concern for the coalition led by Prime Minister Giuseppe Conte. Interior Minister Luciana Lamorgese blamed “uncontrolled flows for creating serious problems in national health security” (*Corriere della Sera*, 28 July 2020). Therefore, alongside more general provisions that considered migrants as part of the resident population, policy measures were also taken to restrict mobility, with borders continuing to be a “battlefield” (Ambrosini, 2021a) between different forces.

This article analyses policy measures introduced by the Italian government during the first wave of the pandemic that were aimed at preventing infection and reducing the impact of COVID-19 among the population that directly or indirectly affected settled and arriving migrants. These measures addressed two emergencies: the spread of COVID-19 that hit the resident population hard, regardless of origin or nationality, and the workforce shortages in some key economic sectors with a high number of irregular migrant workers. The former aimed at containing the spread of the virus (sections 4 and 5) and targeted foreigners already residing in Italy (section 6) as well as irregular migrants arriving along the Mediterranean route; the latter aimed at addressing workforce shortages as a result of borders that were closed to external seasonal migration. Through an analysis of these measures and the rationale behind them, this article aims to contribute to the growing literature on migration and migrant policy during the pandemic and its impact on migrants and foreign populations.

The structure of the article is as follows: the second section surveys the recent literature on migration policy in the pandemic world; the third section explains the methodology used to collect the data for this article. Section 4 to 6 present the main findings: the fourth section examines how the measures taken by the government to contain the virus among the resident population affected foreigners residing in Italy; the fifth and sixth sections focus respectively on quarantine ships as a border closure device for dealing with the pandemic emergency and the regularization of undocumented migrants to address workforce shortages in certain economic sectors. Section 7 critically discusses the main findings of the article.

## Migration, Migrants, and COVID-19: Managing Borders in a Pandemic World

While there are no signs at the moment of an “end to the age of migration” (Gamlen, 2020), the impact of the pandemic on mobility during its first wave in 2020 was enormous. Hundreds of thousands of flights were cancelled, strict new international regulations were enforced, millions of travellers — including migrant workers, transnational commuters, frequent flyers and tourists — were stranded. The EU was also profoundly affected by the outbreak of the pandemic. Although each country took different measures to contain and restrict mobility, the closure of many transit locations, including ports, airports, and land borders, with other restrictive measures, reduced the numbers of arrivals (European Asylum Support Office, 2020; Sanchez and Achilli, 2020), with even Schengen Area EU members reimposing border controls on their neighbours (Benton et al., 2021). A 10% decrease in the number of irregular border crossings to the EU (114,300 in the period January–November 2020) was observed compared with the same period in 2019 — the lowest level in the last six years, although with significant regional variations. While the number of irregular arrivals decreased along the Eastern Mediterranean route (−74%, 19,300), the Central Mediterranean route saw a significant increase (+154%) compared with the same period in 2019 (European Commission, 2021).

The unparalleled character of the pandemic and its consequences on mobility has exercised migration scholars with regard to effects on migration governance and consequences for migrants’ integration and health. According to Newland (2020, p.1) “the governance of international migration is likely to change substantially, in ways comparable to or even greater than the changes that came about after the September 11, 2001, terrorist attacks”. There is the prospect of a shift from cooperation, as envisaged by the Global Compact signed in 2018, to a more individualistic approach, with national governments reclaiming their prerogatives. Other scholars suggest more cautiously that future changes will be characterised by continuity and novelty (Meer et al., 2020), and that measures to regulate mobility will be consolidatory, rather than producing a radical departure from existing securitarian policies (Mezzadra and Stierl, 2020; Tazzioli and Stierl, 2021). In an attempt to hypothesise the possible effects of COVID-19 on migration governance, Cesareo (2021) identifies two possible scenarios. One is positive, characterised by increased solidarity and a renewed humanitarianism. The other is negative, defined by further border closures as demanded by the so-called sovereigntist movements. The link observed between the fast circulation of the virus and global integration (Sirkeci and Yüceşahin, 2020) and the racialisation of the disease generated by the anti-migrant rhetoric of political elites (Perocco, 2021; Reny and Barreto, 2020) provides national and supra-national authorities with opportunities to tighten already-restrictive policies (Clissold et al., 2020; Elias et al., 2021) and ultimately introduce practices of both internal and external bordering. By bordering (Yuval Davies et al., 2019), I mean all those policy measures that aim to govern human mobility and define spaces of belonging take place within, outside, and along official state borders. These measures ‘demarcate categories of people so as to incorporate some and exclude others, in a specific social order’ (Guentner et al. 2016, 392).

Signs of bordering were visible during the first wave of the virus and included border closures, quarantine for arriving migrants, expulsions for the undocumented, and lockdowns of migrant worker communities and refugee camps (Ferrero and Roverso, 2021; Spada, 2021). Several scholars have noted how more stringent policies have mostly affected asylum seekers and refugees, either by ceasing asylum procedures and making it more difficult for migrants to apply for asylum or by closing ports to SAR operations at sea (Guadagno 2020). While these latter measures are not explicit refoulements, they prevent potential asylum seekers from registering at borders (Meer et al., 2020). The pandemic has provided an “excuse” (Stierl and Dadusc, 2022) for sharpening repressive logic and practices that disrespect human rights (Spada, 2021), for downsizing the action of care within humanitarian spaces and favouring traditional securitarian solutions (Sanò and Firouzi Tabar, 2021). In Italy, the focus of our article, this has implied a double emergency for reception, where the practices and procedures of “care,” “cure,” and “control” intertwined and alternated in the context of the pandemic; while, with regard to the EU as a politically and economically integrated unit, the outcome has been a transition from a hostile to an unsafe environment: “Where the former openly declared the manufacture of conditions so adverse as to prompt unwanted individuals to leave, or not even come in the first place, the latter has justified ‘keeping them out’ or ‘containing them elsewhere’ in the name of protecting migrants from the rampant pandemic that has made Europe itself unsafe” (Tazzioli and Stierl, 2021, 78). Similarly, Crawley et al. (2018) argue that migration controls and restrictions have increased inequalities and negatively impacted on migrants’ access to services.

In Italy, the measures taken by governments to reduce mobility affected some categories of migrants in particular, adding to existing barriers to health services and socio-economic inequalities (Perocco, 2021). Data collected during the first phase of the pandemic showed that the risk of hospitalisation and admission to intensive care was higher among the foreign population as a result of delays in diagnosis and reduced accessibility to health services (Geraci et al., 2021). Data also showed how existing barriers to health services for the foreign population, driven by cultural and status issues, weakened measures for containing the virus, and how COVID-19 intersected with and exacerbated pre-existing inequalities (Donà, 2021; Guadagno, 2020). The intersection of inequality and pandemic was particularly true for those migrants with irregular status who were more reluctant to ask for medical assistance. The combination of lockdown measures taken by central government and the uncontrolled proliferation of regional and municipal ordinances that closed access to parks, green areas, and cycle-pedestrian routes — usually refuge areas for those who are temporarily homeless — made the existence of undocumented migrants and asylum seekers expelled from the reception system even more difficult (Ferrero and Roverso, 2021). Other categories of migrants whose conditions exacerbated the effects and consequences of the pandemic were those who worked irregularly, particularly in the agricultural sector, but also as care workers in private households, and those who lived in informal settlements, lacking sanitation, and hygiene facilities with minimal healthcare services (Della Puppa and Sanò, 2021; Filippi and Gilberti 2021; Perocco 2021).

The closure of borders and other state-imposed restrictions to make borders more “secure” has neither stopped irregular migration nor eliminated the demand for smuggling. Rather, in this situation of closed borders and the forced absence of NGOs, journalists, rescue services, and public authorities (Perocco 2021) migrations have become even more irregular, increasing the demand for smuggling services — and consequently their costs — and placing migrants and asylum seekers at higher risk (Sanchez and Achilli, 2020). While some smugglers were initially reluctant to operate out of fear of contagion, with a resultant pause in irregular movement, this withdrawal from operation was only temporary (Bird 2020). What happened along the central Mediterranean route to Italy in 2020 exemplifies this process. Independent journeys on small boats increased dramatically during the most acute phase of the pandemic, particularly from Tunisia, but this growth was only temporary. In 2021, the smuggling industry resumed and departures from Libya organized by smugglers’ organizations again formed the majority of cases (Montagna, 2021).

As this brief review shows, the literature agrees that the pandemic has produced a tightening of borders, increasing the vulnerability of the foreign population and the precarity of migrant journeys. The following sections will analyse the case in Italy, one of the main EU destination countries over the last few years.

## Methodology

This article aims to understand the impact of the lockdown measures taken by the Italian government on arriving and settled migrants and is based on 27 in-depth and open-ended interviews carried out during different waves of the pandemic between April 2020 and late spring 2021with individuals from NGOs and humanitarian and medical aid organisations, lawyers, journalists, a former member of parliament, Red Cross volunteers, and local stakeholders from different parts of the country. Through my fieldwork I tried to understand the aims of these measures and whether they were influenced by the current climate towards migration and therefore whether the pandemic was becoming a device for further securitarian policies.

The sample was chosen according to the skills and experience of each participant; access was possible because I had met and interviewed some during previous research and as a result of the typical snowballing method. The sample can be divided into three main groups, according to the kind of information I aimed to collect and the focus of the interviews. The first group was made up of practitioners working for humanitarian associations, lawyers, and unionists who campaign on migrant labour issues. The aim of these interviews was to understand in more detail the measures taken (or not) by the government and the impact of such on the foreign population. These interviews were mostly carried out during the first wave of the pandemic (spring–early summer 2020), and the questions asked of this group concerned the measures taken by government to minimize the impact of the pandemic and lockdown on incoming migrants and the foreign population living and working in Italy.

The second group was interviewed a few months after the quarantine ships started to operate. It consisted of practitioners in humanitarian organizations based in Sicily, where most of the ships were docked, who were collecting information about quarantine ships and campaigning against their use, as they were considered in violation of human and health rights. Therefore, the questions focused on the quarantine ships, and whether interviewees had visited them or met the migrants aboard. I also asked about their campaign and the reason for their opposition to the ships. Finally, the third group consisted of seven Italian Red Cross volunteers whom I interviewed with the aim of understanding their experiences on quarantine ships. Questions focused on the positions they held, how they were recruited, the length of notice they were given before boarding, whether they had any specific training, and other practical issues regarding life on quarantine ships, including the kinds of relationships established with the migrants. These were only indicative questions as I always tried to establish some sort of dialogue rather than rigidly sticking to a fixed schedule during my interviews, which ranged in length from 20 to 90 min. While some interviews were conducted in person once lockdown was eased between May and October 2020, much of the fieldwork has utilised the platform Zoom. The names of the participants have been anonymised.

This article is also informed by an extensive analysis of grey literature, including pieces of legislation, official government documents, blogposts from activists, migrant associations, and lawyers, and on a review of the existing literature.

## The COVID-19 Emergency and the Impact of Anti-pandemic Measures on the Foreign Population During the First Wave of the Pandemic

Lockdown measures aiming to mitigate — and eventually interrupt — the spread of COVID-19 proliferated during the first wave of the pandemic. Between January and April 2020, a total of 246 different COVID-19–related measures and acts were issued by the Italian government, an average of 61.5 per month: there were 11 COVID measures issued in January, 67 in February, 103 in March, and 65 in April (Openpolis, 2022). These measures involved the closure of public offices, including prefectures, courts, police stations, many of the other offices dealing with migration issues, and many private institutions, such as banks, insurance companies, driving schools, bars and restaurants, and nearly all those related to hospitality. Industrial activity also ceased, with the exceptions of agriculture, the food industry, and some manufacturing related to essential services. All people who resided in Italy had to stay home, could not leave their place of residence or their region, were only allowed outside in cases of proven necessity, such as food shopping, work, dog-walking, and exercise, and had to follow rigorous procedures against contamination and strict hygiene measures, such as the obligations to wear protective masks and gloves and to maintain physical distance.

The effects of these provisions on the foreign population differed according to status (i.e. undocumented, asylum seekers, and regular) and housing situation (i.e., reception, informal camps or other accommodation). As a lawyer of the migrant advocacy group *Associazione diritti per Tutti* said, “not all had necessarily a negative impact on the foreign population. I would say the opposite”. For example, administrative and legal documents issued by the Italian authorities for documented migrants, such as residence permits and identity documents, that were due to expire during the lockdown were extended until the end of the year, when, it was believed, the emergency would end. Similarly, reception projects for asylum seekers were automatically prolonged for 6 months since new tenders could not be issued (Codini, 2021).

Other effects were less favourable, particularly to asylum seekers and those waiting for a response to applications to settle in Italy. The suspension of measures concerning administrative proceedings, for example, delayed the granting of citizenship to those who applied for it. Similarly, all appointments in the process for reunification permits were postponed, thus deferring the possibility of migrants reuniting with family members (Zorzella, 2020), all judicial proceedings relating to international protection and hearings before territorial commissions for the recognition of the right of asylum were suspended. While the Interior Ministry promised to keep immigration offices open and allow asylum seekers to apply for international protection, this happened only in theory. As a lawyer from the *Associazione per gli Studi Giuridici sull’Immigrazione* explained:

“We received numerous reports of people unable to access immigration offices to formalise the application for international protection because they were closed, with the consequent exclusion from the reception system, and of people who were on the street and who did not have available accommodation and were therefore at risk of being subjected to expulsion orders.”

One process that continued was the issue of expulsion papers. During the first phase of lockdown, some categories of migrants were more vulnerable to being stopped and identified by the police, namely those who were rendered irregular by the Salvini Decrees of 2018 and 2019, which abolished humanitarian protection and redesigned the reception system, throwing thousands of people into informal settlements and squats, or onto the streets to sleep rough (Zorzella, 2020). According to an activist from the *Associazione Diritti per Tutti*:

“In this situation, former seekers of international protection who were deported or simply expelled from reception found themselves on the streets, homeless. With the lockdown and no one around, it was super-easy to identify them and provide them with a deportation order”.

Although these expulsion decrees did not become actual repatriations until June, when lockdown was eased, they remained punitive measures that contributed to making undocumented migrant lives even more precarious and risked their settlement in Italy.

## The Pandemic Emergency, Border Closures, and Health Surveillance on Quarantine Ships for Arriving Migrants

As discussed in the first section, globally the pandemic became an opportunity for activating bordering measures and strengthening external borders with the aim of controlling mobility and irregular entries. Italy was no exception in these respects. As a member of *Borderline Sicilia*, a monitoring association based in Sicily, argued, “the pandemic became an opportunity, a sort of laboratory, I would say, for introducing new security measures and experiment new forms of border control”. In this respects, two key policy measures were passed during the first phase of lockdown in April 2020, both aimed at tightening border controls. First, government passed the *Decreto Interministeriale* n.150, 07 April 2020 (Gazzetta Ufficiale, 2020a), declaring that, because of the national health emergency deriving from the spread of the COVID-19 virus, Italian ports could no longer be classified as places of safety for rescue operations carried out by naval units flying a foreign flag (i.e., SAR ships) outside territorial seas. The decree equated Italian ports with countries at war where respect for human rights is not guaranteed. Second, this provision paved the way for the *Decreto n.1287 del 12 aprile 2020* establishing the organization and placement of quarantine ships for carrying out the period of health surveillance for all migrants rescued at sea or arriving after autonomous landings. With this measure, government decreed the use of quarantine ships for people rescued at sea during SAR operations by foreign ships outside Italian territorial seas if it was not possible to find a disembarkation points in other safe countries (Dipartimento della Protezione Civile, 2020).

Initially, the establishment of quarantine ships was to be temporary, an emergency measure aimed at managing the risk (Beck, 1992) created by the pandemic. However, the increasing number of arrivals and the increase — however slight — of positive cases in reception, accompanied by protests by local communities against COVID centres often led by anti-migrant parties, changed the original plan. Between early July and September, several calls for tender were issued and, by mid-September, Italy had rented seven quarantine ships from private company *Grandi Navi Veloci*.

Although the actual number of incoming migrants housed on the quarantine ships during the time they were in use is unclear — the government has never provided official data on this — press sources reported an estimated 3000 people held in quarantine by mid-September 2020 (Vita, 2020). They included migrants who disembarked independently at Lampedusa and other landing points in southern Italy, but also those with regular status housed in reception centres on the mainland, in breach of the decree. Until the government changed its policy in October, children and unaccompanied minors, and migrants already in reception were also subjected to this treatment.

Migrants were immediately boarded after their disembarkation from SAR ships on land — usually in Lampedusa — and held until the quarantine was complete or they tested negative. During their stay on these “floating hotspots”, arriving migrants had no access to legal information, very little medical aid, no clear information regarding their futures. There was no capacity for identifying vulnerable cases, such as victims of violence and trafficking (Associazione per gli Studi Giuridici sull’Immigrazione, 2021a). After the quarantine period, migrants were taken to the mainland to be transported to detention centres and given an expulsion decree if they were to be deported or taken to reception facilities if they were considered deserving of asylum. The decisions on whether migrants deserved to apply for asylum were mostly based on nationality, rather than on a case-by-case basis, with no chance for arriving migrants to present and discuss their reasons. Informants interviewed for this study reported that all Tunisians, who over the previous few years had become the main national group landing on Italian shores, were automatically provided with a decree of expulsion once quarantine was over, and no preliminary investigation was conducted into their reasons for seeking asylum in Europe. As a volunteer for the NGO Borderline Sicily said:

“The only preliminary investigation is that you are Tunisian and you are Tunisian because you told me so. I give you the expulsion decree because there are no places in the repatriation centres. There is this further qualitative leap in the abuse against certain categories of people.”

The use of quarantine ships was discontinued as of 31st May 2022, 2 months after the end of the state of emergency. Although it is too early to say whether this measure was an experiment to introduce a new method of receiving and managing migratory flows and will therefore be revived, perhaps under a different guise, or whether it will disappear permanently along with the pandemic emergency, their use raises a number of concerns. Firstly, they were ill-suited for addressing the range of issues related to the mental and physical health of vulnerable people and those arriving in Italy after traumatic experiences in places of captivity in Libya or at sea. According to the information collected for this article and presented in several humanitarian NGO and media accounts, the quarantine ships lacked specialised and skilled figures, and psychological and physical health provisions were dramatically under-resourced if not entirely absent (MicroMega 2021, 9 November 2021). A study based on 82 interviews with foreign citizens held on the quarantine ships during the first wave of the pandemic reported inadequate living conditions and a lack of medical-psychological assistance (Associazione per gli Studi Giuridici sull’Immigrazione, 2021a). Several fatal incidents took place during this period, confirming the precarious legal and medical situation. On 20th May, 28-year-old Bilal Ben Massaud jumped into the sea to swim to the coast and drowned. On 15th September, 17-year-old Abdallah Said died of tuberculous encephalitis at Cannizzaro hospital in Catania, where he had been transferred after a period of isolation on a quarantine ship. At the beginning of October, 15-year-old Abou Diakite died following an emergency hospitalization that was carried out only after many days of isolation on a quarantine ship. Secondly, the quarantine ships had a discriminatory nature, since no other category of people was subjected to such restrictions or physically prevented from leaving a place under surveillance by security agents. While the resident population who came into contact with the virus had the option of fiduciary quarantine, and the crews of SAR ships were required to quarantine on board — even after having no contact with the virus — neither travellers from other countries nor seafarers on commercial shipping were required to self-isolate.

Thirdly, the quarantine ships created a health security-migrant nexus. They racialised the virus and, by doing so, stigmatised arriving migrants as spreaders of the virus and a threat to national health security (O’Brien and Eger, 2020). Migrants have been historically stigmatised as carriers of disease, particularly in recent years with the growth of migratory flows (Ambrosini, 2021b). At the start of the COVID-19 pandemic, the virus was associated with Chinese people and migrants, increasing hostility and physical attacks towards anybody coming from the Far East (Reny and Barreto, 2020). With the use of quarantine ships the State constructed arriving migrants as a distinct category by identifying them as potential *untori*^1^ (plague-spreaders) to be kept at a distance and subjected to exceptional bordering measures.

## Workforce Shortages and the Regularization of Undocumented Migrant Labourers

Other areas of policy intervention concerned the health emergency in informal settlements such as Rosarno Calabro in Calabria and Capitanata and Borgo Mezzanone in Apulia, and the workforce shortages in some key sectors, such as agriculture and home care. During the first days of lockdown, it was clear that in informal settlements, mostly composed of shacks without running water or sanitation, there was “an emergency within the emergency” (Zorzella, 2020: 3), and instructions issued by the government to stay at home or to wash hands were unrealistic. Public authorities carried out very little preventive intervention, such as medical aid, information or health screening, and often the only support for alleviating vulnerability was provided by NGOs in mobile clinics (Tagliacozzo et al., 2021). Migrants themselves tended to underestimate the virus, either out of ignorance of the symptoms, or from fear of losing their jobs (Medici per i Diritti Umani, 2020). The government addressed these concerns by adopting some limited measures. The most notable was the acceleration of the start of “Più SUPREME”, an EU programme led by the Italian Ministry of Labour and Social Policies involving five southern regions: Apulia, Sicily, Campania, Calabria, and Basilicata. When the programme was outlined in 2019, its aim was to combat labour exploitation in agriculture and to intervene with a series of integrated actions of assistance, treatment, protection, and prevention in favour of third-country nationals in a vulnerable situation (Camera dei Deputati, 2020). With the pandemic, part of the funding was spent on preventing the spread of the virus through doctors, nurses, volunteers, and intercultural mediators assisting migrants with rapid tests and medical examinations, information about the virus, and the distribution of preventive kits and food packs (Medici per i Diritti Umani, 2020; Tagliacozzo et al., 2021).

Labour conditions were also heavily affected during the first wave of the pandemic. As several studies conducted before the COVID-19 outbreak have shown, migrants’ irregular status and working contracts exacerbated exploitative conditions in all economic sectors. With the pandemic and the subsequent lockdown and border measures exploitation of migrants worsened, particularly in sectors such as agriculture. As stated by a member of the union *Confederazione Generale Italiana del Lavoro*, this happened for several reasons:

“The absence of controls and an acceleration in demand for fruit and vegetables has produced not only a lowering of protection and income or wages, but also a significant increase of about 12–16% in work intensity. They not only earn less, they work longer, and they work with much greater intensity”.

As this issue was becoming more evident, NGOs, humanitarian associations and unions called for major government intervention in those areas where exploitative working conditions were worsening due to the pandemic and lockdown measures (Trucco, 2020). However, the government’s attention to intervene in the agricultural sector was drawn by appeals from agricultural entrepreneurial associations, rather than out of a desire to assert human and labour rights. The agribusiness sector was alarmed that a prolonged blockade and borders closed to seasonal migrants would produce a potential shortage of about 200,000 workers if the pandemic worsened. On the other hand, Secretary of Agriculture Teresa Bellanova, a former farmer worker and unionist, also warned about the condition of those people who ‘already work on our territories and live in informal settlements and are underpaid, often inhumanly exploited, and even more exposed to health risks and hunger’ (*La Repubblica* 4 May 2020). According to Secretary Bellanova the regularization of these workers was a matter of urgency.

After gruelling negotiations within the coalition, with the centre-left parties more open to wider amnesty and the 5 Star Movement in favour of more restrictions, and after overcoming opposition from the right-wing parties, the government passed the *Decreto Legge 34* (Gazzetta Ufficiale, 2020b) on 19th May 2020, known as the “Relaunch” decree. This comprised economic measures, including the regularization of Italian and foreigner workers in selected industries such as agriculture, fishing, and assistance for the elderly or people with disabilities, aiming to support the country in a very difficult moment. As for undocumented migrants, three paths were contemplated. The first allowed employers to regularize foreign workers provided they were employed in one of the aforementioned sectors and were already in Italy by 8th March, 2020, the day the national emergency was declared, and had received residence permits. The second allowed foreigners whose residence permits had expired after 31st October, 2019 to apply for temporary residence permits with the possibility of these being converted to a residence permit for work. While these two pathways relied on the activation of migrant workers’ employers, the third was a 6-month temporary permit that could be turned into a work permit if an employment relationship was established before its expiration. The decree did not introduce any specific measures for supporting undocumented migrants during the pandemic (Carlotti, 2020).

The outcome fell below the expectations of those who wanted a generalised and more substantial regularization. By the 15th August deadline, only 207,000 migrants had applied, one-third of the estimated 600,000 irregular migrants in Italy in 2020 and slightly less than the 220,000 expected by the government. Only 15% of applications were from agricultural workers, the main aim of the regularization as the sector most at risk of workforce shortages, while the vast majority (85%) were from care workers (Pasini and Regalia, 2021). The lower number of applications may be due to the difficulties and barriers involved in the application process and the inconsistencies in the decree. Moreover, relying on employers to apply for regularization may have limited migrant workers’ access to the process, especially considering that the sectors involved in the amnesty are characterized by high levels of informality (Associazione per gli Studi Giuridici sull’Immigrazione, 2021b). It is arguable whether this amnesty was a success or a failure. It certainly represented an exception in an international landscape wherein no country implemented extraordinary regularization measures in the wake of the pandemic (Ambrosini, 2021b).

## Discussion

The policy measures related to migrants and migration documented in this article aimed to address two emergencies: the spread of the virus and the workforce shortages in sectors with high levels of labour migration between February and early autumn 2020. The first set of measures concerned migrants as a component of the population residing in Italy and were aimed at containing the virus. During the first wave of the pandemic and the strictest phase of the lockdown the rules for migrant residents and the rest of the population were the same, including mask-wearing, maintaining social distance, the prohibition of gatherings, and staying home except in cases strictly indicated by the legislator. The impact of this apparently undifferentiated blanket of measures, however, varied enormously between different groups. The instruction to “stay at home” made migrants who were sleeping rough and those who lived in overcrowded informal settlements more vulnerable to police control, expulsions and the risk of infection. The closure of offices to the public also had varied impact, with some groups more penalised than others by delays in the assessment of asylum applications or family reunion, increasing uncertainty among asylum seekers and migrants’ families. In addition, as a lawyer from the *Associazione per gli Studi Giuridici sull’Immigrazione* explained, “Measures relating to the spread of Covid-19 arose from a common sense logic but then were managed by offices that have to handle them in conditions of structural difficulties, organizational problems, and lack of resources. Some of these problems are structural, others due to the uncertainty caused by the pandemic and the lockdown.”

Another measure aimed at addressing the spread of the virus while managing migration was the introduction of a “floating” hotspot system, in which migrants arriving along the Central Mediterranean route were confined and identified in a similar way as in the on-shore hotspots envisioned by the 2015 European Migration Agenda (D’Angelo et al. 2017). Using the pandemic emergency as a pretext, this measure contributed to the securitarian tightening of external borders in line with the bordering practices that characterise Italian and EU migration policy. While the quarantine ships were a new tool for managing EU borders — the first time an at-sea identification system took place in the EU — this floating system of identifying arriving migrants did not represent a departure from the policies that characterise the EU approach to migration. It is questionable whether it was “a device for the externalisation of borders” (Spada, 2021: 11) or more simply a device designed to securitize Italy’s borders, since quarantine ships were still part of Italian territory. Certainly, they sharpened bordering repressive practices and enhanced the disrespect for human rights while introducing elements of novelty to the ways borders are securitised (Ibidem: 11).

The other emergency was the workforce shortage as a consequence of the lockdown and borders being closed to seasonal workers. This was addressed by a measure aiming to regularize undocumented migrants and irregular workers and attempting to respond to demands from agribusiness entrepreneurial associations and government ministers regarding a shortage in some sectors of the labour market. Like other past amnesties in the regularization of undocumented immigrants in Italy (Montagna, 2013), this was also conditioned by the highly politicized and divisive migration debate. While this regularization did not meet demands from human rights and medical care associations for a more general amnesty, it is not clear whether it fulfilled the expectations of business associations and the government (Dal Zotto et al., 2021). As of 31st December 2020, in fact, of the more than 207,000 applications submitted by employers for an irregular employment relationship or the establishment of a new relationship with foreign citizens, only 1480 residence permits were issued on this basis, less than 1% of the total (*Avvenire* 4 March 2021). As a result, by spring 2021, agricultural entrepreneurial associations still warned about a possible workforce shortage in their industry (*La Stampa* 19 April 2021).

## Conclusion

To conclude, this article has considered measures that sought to address the two emergencies, the spread of the virus and the workforce shortages that arose in Italy in relation to the foreign population already in the country and to incoming irregular migrants along the Central Mediterranean route. It has showed the coexistence of two different approaches. On the one hand, these measures were affected by the unexpected situation the country experienced during the health emergency. Governments across the globe, including Italy, were dealing with an unprecedented situation that caused hundreds of thousands of deaths, and an economic collapse during which measures had to be invented from day to day with limited resources available and organisational difficulties. Within this context migrants were experiencing “an emergency within the emergency”, where the impact of an unexpected and dramatic situation overlapped structural disadvantages: family reunions were delayed for the closure of public offices, asylum applications were stopped, etc. On the other hand, in relation to the migrant phenomenon, these measures have followed the traditional emergency and securitarian approach of Italian governments, which seeks to combine border security with constant labour turnover in some crucial industries such as agriculture, care for the elderly, and cleaning. Both the quarantine ships and the agricultural labour shortage decree followed this pattern: secure the borders but providing labour force in key sectors which were paying for the lockdown measures. Thus, the intertwining of the pandemic and immigration has reinforced the inclination towards further bordering, in an attempt to protect the insiders (domestic natives) from the outsiders (foreign natives), without forgetting the labour needs of companies in the agricultural industry.

## Footnotes

1 *Untore* is a derogative Italian term used to indicate those believed to have voluntarily spread the plague by spreading special ointments in public places and was popularised by Alessandro Manzoni in his novel set in the seventeenth century, *I Promessi Sposi* (The Betrothed).
